# Mississippi Valley Dental Society

**Published:** 1879-04

**Authors:** 


### MISSISSIPPI VALLEY DENTAL SOCIETY.

The thirty-fifth annual meeting of the Mississippi Valley Dental Society, was held on March 5th, 6th and 7th. According to the annual custom in this society, a good time was enjoyed by those present. A much larger number was in attendance than usual. The subjects presented and discussed were interesting, and elicited many new thoughts.

No attempt will now be made to give a synopsis of the discussions; these, with the proceedings, will be published in the REGISTER.

Many new faces appeared, and some were missed who have usually been present in former years. Those who were the active members thirty years ago are becoming less and less as the years go by, but their places are filled by new recruits.

This, the oldest dental society in existence, gives evidence of vigorous life, and a prolonged course of usefulness.

Long may it live.

